# Cooperation on Interdependent Networks by Means of Migration and Stochastic Imitation

**DOI:** 10.3390/e22040485

**Published:** 2020-04-23

**Authors:** Sayantan Nag Chowdhury, Srilena Kundu, Maja Duh, Matjaž Perc, Dibakar Ghosh

**Affiliations:** 1 Physics and Applied Mathematics Unit, Indian Statistical Institute, 203 B. T. Road, Kolkata 700108, India; jcjeetchowdhury1@gmail.com (S.N.C.); srilena93@gmail.com (S.K.); 2 Faculty of Natural Sciences and Mathematics, University of Maribor, Koroška cesta 160, 2000 Maribor, Slovenia; maja.duh3@um.si (M.D.); matjaz.perc@gmail.com (M.P.); 3 Department of Medical Research, China Medical University Hospital, China Medical University, Taichung 40402, Taiwan; 4 Complexity Science Hub Vienna, Josefstädterstraße 39, 1080 Vienna, Austria

**Keywords:** cooperation, interdependent networks, mobile agents, prisoner’s dilemma, snowdrift game, game theory, mobility, rational agents

## Abstract

Evolutionary game theory in the realm of network science appeals to a lot of research communities, as it constitutes a popular theoretical framework for studying the evolution of cooperation in social dilemmas. Recent research has shown that cooperation is markedly more resistant in interdependent networks, where traditional network reciprocity can be further enhanced due to various forms of interdependence between different network layers. However, the role of mobility in interdependent networks is yet to gain its well-deserved attention. Here we consider an interdependent network model, where individuals in each layer follow different evolutionary games, and where each player is considered as a mobile agent that can move locally inside its own layer to improve its fitness. Probabilistically, we also consider an imitation possibility from a neighbor on the other layer. We show that, by considering migration and stochastic imitation, further fascinating gateways to cooperation on interdependent networks can be observed. Notably, cooperation can be promoted on both layers, even if cooperation without interdependence would be improbable on one of the layers due to adverse conditions. Our results provide a rationale for engineering better social systems at the interface of networks and human decision making under testing dilemmas.

## 1. Introduction

Evolutionary game theory [1,2,3,4,5,6,7] gained a widespread recognition due to its applicability in various interdisciplinary domains ranging from biological to social sciences, economics to psychology, and mathematics to physical sciences [8]. Due to the Darwinian theory of the survival of the fittest, the emergence and persistence of cooperation [9] among unrelated selfish individuals is a fundamental challenge in nature’s evolution. How to achieve global and individual optima of cooperation in a competitive environment is the main interest of mathematicians, biologists, physicists and social scientists. Although cooperation is a costly move, it can be observed in many real situations. A ‘helper’ bird [10] often takes care of an individual other than its mate. The simple organisms [11,12] like ants and bees also exhibit fascinating spatial cooperative behaviors. Paradigmatic examples are the Prisoner’s Dilemma (PD) [13] and the Snowdrift game (SD) [14]. These are often explored theoretical frameworks [15,16,17,18,19,20,21,22,23,24,25,26], where cooperators bare a cost for the collective well-being while defectors do not contribute whilst still enjoying the same benefits.

On the other hand, network science [27,28,29,30], a new discipline emerging in the 21st century, reveals many unanticipated collective phenomena ranging from the internet to sociology, biochemistry to brain science, to name but a few. Interestingly, the effect of network reciprocity on the evolutionary game [31] has been identified as in early as the 1990s by Nowak and May. In the last decade, this Nowak–May model has been extended under several conditions to maximize cooperation [32]. In this context, the introduction of multilayer networks [33,34,35,36,37,38,39] opened up a new direction to explore [40], which helps to understand how information available in one network layer affects the behavior of the other network layer [41]. Multilayer network already captures the spotlight to evaluate the impact of network of networks on the evolution of cooperation [42,43,44]. Although, most of the previous studies incorporated the same game-theoretic models in all layers. But, in reality, individuals can delineate behavioral heterogeneity [45,46,47,48]. Such diversity can be reflected in terms of aspiration level [49], personal learning capability and so on. Szabó et al. [50] discussed the effect of inhomogeneous strategy transfer capability, which promotes cooperation within a moderate density of influential players in the spatial prisoner’s dilemma game model. Zhu et al. [51] also investigated the influence of two types of layers on the public good games and they found that their heterogeneous strategy updating process greatly enhances the evolution of cooperation in the structured population under the intermediate fraction of influential players.

Recently, the attention of the researchers has been shifting towards the consequences of mobility of individuals [52,53,54], particularly to those scenarios where the spatial structure is known to hinder cooperation. Although the effect of mobility in the context of evolutionary game theory seems to be an incalculable puzzle, as movements in human beings and living organisms are often modeled as a random walk [55]. The mechanisms of these random walks are different solely based on the goal of the movement. ‘Move after partner defects’, this strategy can outperform other complex strategies under a certain number of suitable conditions [56]. Vainstein et al. [57] proposed an “always-move” strategy in a diluted Nowak and May spatial Prisoner’s Dilemma model. They found that their strategy, under the availability of enough free spaces, can increase cooperation compared to the static (non-mobility) case for a range of parameter values. The role of different movement strategies is surveyed in [58].

In this present article, we want to explore the interplay between migration of individuals and interdependence between the multilayer network in the evolution of cooperation. For this purpose, we consider an interdependent network, where each layer corresponds to different types of games. In the existing literature, an interdependent network is defined as a multilayer network consisting of dependency interlinks (not physical connections) between the nodes in several networks and each layer represents different types of nodes [40]. We propose a migration scheme oriented goal, solely based on the principle of maximizing pay-offs (see Section 2 for details). Instead of the random diffusive re-location policy, our work aims to investigate how this new migration strategy affects the organization of cooperation in an interdependent network of different game playing layers. Interlinks between layers are established probabilistically, which enables people to update their respective strategies occasionally from long distant neighbors. The remaining part of this article is organized as follows. In Section 2, the preliminary ingredients of the paper, the strategy updating algorithm and the considered evolutionary games are thoroughly discussed. Section 3 is devoted to the presentation of numerically simulated results, and finally, we conclude our findings with their potential implications in Section 4.

## 2. Methods

### 2.1. Algorithm for the Strategy Updating

The employed strategy updating algorithm is as follows. We consider *M* number of layers, and each layer is a square lattice of size L×L with periodic boundary condition. Initially, $f_{0}$ fraction of free spaces are considered in all layers. Thus, the number of free lattice points is $N_{f}=\lfloor L\times L\times f_{0}\rfloor$, and consequently, the number of occupied lattice points is $N=L\times L-N_{f}$. Out of *N*, 50% of the individuals are randomly designated as cooperators (C) and remaining individuals are designated as defectors (D). At every iteration step, individuals are updated asynchronously in a random sequential order.

Each randomly selected individual gets an equal opportunity for moving to the eight neighboring cells surrounding it, provided those cells are empty, i.e., not occupied by any other individuals. Moving to any of those vacant cells of the Moore neighborhood of size 3×3, those individuals participate in a fictitious game and calculate the expected returns (payoffs). The payoffs are accumulated due to simultaneous interaction with the players situated in the Von Neumann neighborhood of that lattice point at a Manhattan distance of 1.

With probability (1−r), an individual moves to the site with the highest pay-off and imitates the strategy of the best performing neighbor, if the own pay-off is lower. In the case of more than one cell with the highest payoff, any one cell is selected randomly. If the fictitious payoff is less than the payoff collected in the current position, the individual remains in his/her cell. On the other hand, the individual likes to update its strategy with probability *r*, from a neighbor of the player sitting in the replica position of the opposite layer, provided that cell in the replica position is occupied by some individual. If the replica position is empty, then the strategy of the best performing neighbor within the same layer is copied after the proposed migration and imitation step. If an individual does not have any free spaces surrounding him/her within its own layer, it will not update its strategy at that step.

**Figure 1:**
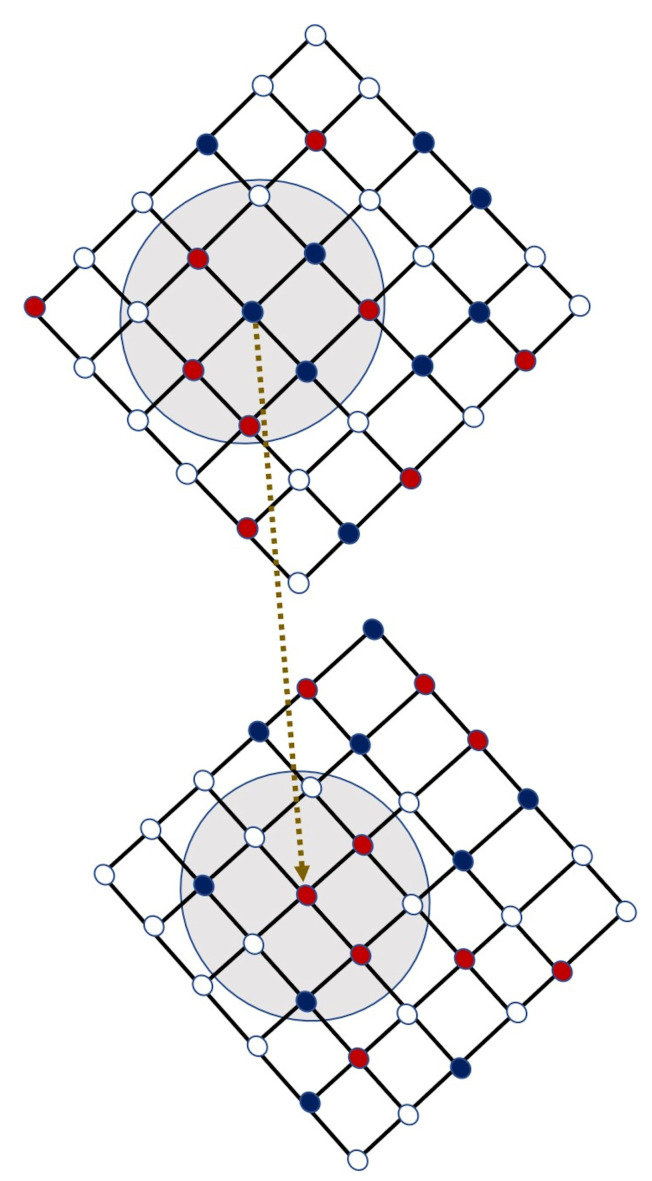
Schematic diagram: a bi-layer interdependent network is considered, where each network is a L×L lattice with L=6. White lattice points symbolize free spaces, red cells stand for defectors and blue points represent cooperators, respectively. Each focal player can move into any of the existing vacant (white) cells within the shaded circular region. $f_{0}=50\%$ free spaces are taken into consideration for this illustration. In case that a player does not find any vacant cell inside its Moore neighborhood, he/she does not update his/her strategy at that step. At any particular time iteration, the players of the first layer update their respective strategies, then at the same time iteration, the players of the second layer get the same opportunity to update their respective strategies.

In Figure 1, a simplified graphic is manifested. In this representation, an interdependent network with M=2 layers is considered. Note that the individuals of the *i*-th layer get the first chance to upgrade their respective strategies at a specific time (iteration) and then, the individuals of the (i+1)-th layer, and so on. For instance, in the schematic picture (Figure 1), layer 1 (upper layer) gets the first chance to update and then the second layer (bottom layer) will be updated at the same time. So, the stochastic interlinks created between two layers are directed in nature. Such an interlink between the nodes $V_{\left(3,3\right)}^{1}$ and $V_{\left(3,3\right)}^{2}$ are shown in the figure, where $V_{\left(i,j\right)}^{\alpha }$ represents the vertex at the (i,j)-th position of α-th layer.

### 2.2. Network and Game-Theoretical Model

We first employ our movement strategy updating policy for M=2 layers of interdependent network. Two distinct 2×2 (two-person) games are considered in two layers. Players in one sub-population follow the PD game, while the SD game is followed by the players in another layer. It does not affect our simulated results that which game model is played in which layer. The general payoff matrix resulting from the interaction between two players is given by

$$\begin{array}{cc} & \begin{array}{cc} C & D \end{array}\\ \begin{array}{c} C\\ D \end{array} & \left(\begin{array}{cc} R & S\\ T & P \end{array}\right) \end{array}$$

in which the entries represent the payoff accumulated by the player in the left. The ordering between the entries of this payoff matrix determines the playing game. These quantities are ranked as *T_SD_* > *R_SD_* > *S_SD_* > *P_SD_* for SD and the PD game is delineated for *T_PD_* > *R_PD_* > *P_PD_* > *S_PD_*. This slight variation in the relative ordering produces a notable change in the game dynamics. Here, the interaction between two defectors results in a punishment *P*, which is clearly worse compared to the reward *R*, gained by two players who choose to cooperate with each other, as *R* > *P* in either of the games. The interaction between a cooperator and a defector produces a sucker’s payoff *S*, for the former while the latter receives a temptation *T*.

Hence, our investigation possesses three different folds and these are (i) interdependence of upper and bottom networks, (ii) migration of individual in a sparse network, and lastly (iii) combination of PD game with SD game. Each of these facts can boost the fraction of cooperation considerably under certain suitable circumstances. However, their cumulative effect is not studied yet. Obviously, PD and SD are two of the possible two-person games to study the effect of heterogeneous strategy updating process. There are several other games [59], like Public goods game, Stag Hunt game, Leader game, Hero game, Avatamsaka game, Anti-Leader game, Anti-Hero game and many more. But, without loss of generality, we choose PD and SD games for their simplicity and enormous applications in biology, economics, ecosystems and sociology [13,60,61,62,63]. Motivated by these facts, already thousands, and possibly millions, of studies have involved dilemma games, including PD and SD games. Particularly, donor-recipient game (DRG) (also known as donation games or mutual aid games), one of the sub-classes of PD games, gains its well-deserved attention due to its applicability in biology and ecosystems [64,65]. DRG is a game structure described by two-parameter benefit, *b* and cost, *c* of cooperation. Nowak [3] proved that there exists a possible universal scaling law, fraction of cooperation = function of $\left(\frac{b}{c}\right)$ for DRG, when considering the major reciprocity mechanism. On the other hand, spatial structure often promotes the evolution of cooperation for PD game [31], but spatial structure is found to reduce the proportion of cooperators for SD games unexpectedly [66], if the cost-to-benefit ratio of cooperation is high. Inspired by all these facts, we choose PD and SD games in both layers to incorporate the heterogeneity in the global interdependent network.

Throughout our study, the lattice size in each subnetwork is taken as L×L, with L=100. All simulations are presented after $t=10^{3}$ iterations with 30 independent statistical realizations (unless otherwise mentioned). The results remain unaltered for any longer iteration length and if averaged over larger realizations. Without loss of any generality, the payoff values are fixed as $R_{PD}=R_{SD}=1.0$, $S_{PD}=S_{SD}=0.0$, $P_{PD}=0.1$ and $P_{SD}=-0.4$. These specific choices of parameters lead to dilemma strength [59,64] of first layer $D_{g}^{1}=T_{PD}-R_{PD}=T_{PD}-1.0$ and $D_{r}^{1}=P_{PD}-S_{PD}=0.1$ and the dilemma strength of the second layer is $D_{g}^{2}=T_{SD}-1.0$ and $D_{r}^{2}=-0.4$. Thus, our chosen games are general, as $D_{g}^{1}\neq D_{r}^{1}$ (unless $T_{PD}=1.1$) and $D_{g}^{2}\neq D_{r}^{2}$ along with $P_{PD}$ and $P_{SD}$ both are non-zero.

## 3. Results

**Figure 2:**
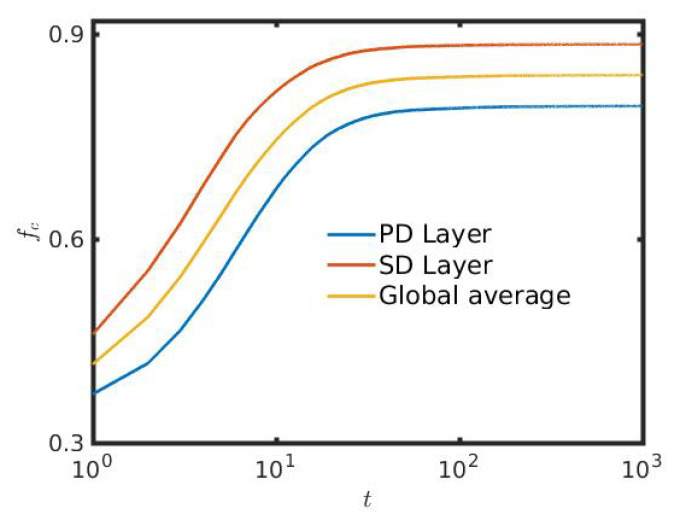
Fraction of cooperators $f_{c}$ as a function of time (iterations), *t*: The parameters are taken as follows: $T_{PD}=1.3=T_{SD}$, $f_{0}=50\%$ and r=0.2. At the initial time t=0, the fraction of cooperators is fixed at 0.5, as all the defectors and cooperators are initially equally distributed. The *x*-axis is given in logarithmic scale. Thus, the value of $f_{c}$ at t=0 is not shown here. PD: Prisoner’s Dilemma; SD: Snowdrift game.

Figure 2 shows the fraction of cooperation $f_{c}$, defined as $f_{c}=\frac{\mathrm{Number}\;\mathrm{of}\;\mathrm{cooperators}}{N}$, obtained from the interaction between players of two different layers, which is almost 80% for the PD layer and exceeds to almost 90% for the SD layer after the initial transient time period. Earlier, Santos et al. [67] suggested a biased imitation strategy, which is found to be environmentally unfriendly for the SD layer, but favorable for the PD layer on interdependent networks. As per the study by Wang et al. [68], also the introduction of interdependence between interdependent networks is found to amplify the hindrance greater in the SD layer, but promotes cooperation in PD layer. This limitation is surpassed by the introduction of mobility in our proposed strategy updating procedure. The initial additional investments to find a better neighborhood in order to gain more ultimately enhance $f_{c}$ of both layers. In order to increase their pay-offs, individuals go for one of the two strategies. Due to the inclusion of mobility in our present study, a defector having cooperative neighbors, leaves those cooperators to two specific circumstances. One possibility is to move away and find a better neighborhood, or alternate their strategy and become defectors. Therefore, a defector can utilize these benefits only for a shorter period of time. This scenario thus resembles a one-sided love affair, where the defectors are always attracted to the cooperators. But the cooperators do not feel the same attraction towards the defectors. In fact, the attraction between the cooperators and defectors is proportional to S+T, which is comparatively low than the mutual affection between two cooperators. A cooperator, having a cooperative neighborhood will always cherish their company and love to unalter their respective cooperative strategies for a period of successive time iterations. The mutual attraction between the cooperators is 2R. Generally, the mathematical inequality 2R>T+S holds for the chosen games PD and SD. To investigate the cumulative effect of our proposed strategy on the global interdependent network, we also plot the global average in Figure 2. The global average is the arithmetic mean of the fraction of the cooperation of both layers. Promotion of cooperation on the entire network is established through the time evolution of global average $f_{c}$.

Also, in the transient of this Figure 2, at around t=2, a notch type behavior is found in all time series. The exact reasoning behind this transient phenomenon is not clear to us. Initially, the global average is decreasing with respect to time *t* and then, depending on suitable choices of other parameters, $f_{c}$ is increasing. Earlier, the role of interdependence between the networks for the optimal promotion of cooperation [69] is investigated and they found similar qualitative time evolution of the fraction of cooperators. The notch type behavior in the time evolution of the fraction of cooperation is also observed in earlier study [70] of the resilience of cooperative behaviors in multiplex networks. Even so, a similar phenomenon is observed in Ref. [49]. Defectors are actually initially getting fare better opportunities in the most early transient stage of the evolutionary process. The initial decimation of cooperators in the preliminary time series reflects the fact that defectors are, as individuals, more successful than cooperators. After this initial downfall of cooperators, the dominance of $f_{c}$ is established with suitable choices of other parameters. This sudden fast change of $f_{c}$ creates that notch like behavior.

**Figure 3:**
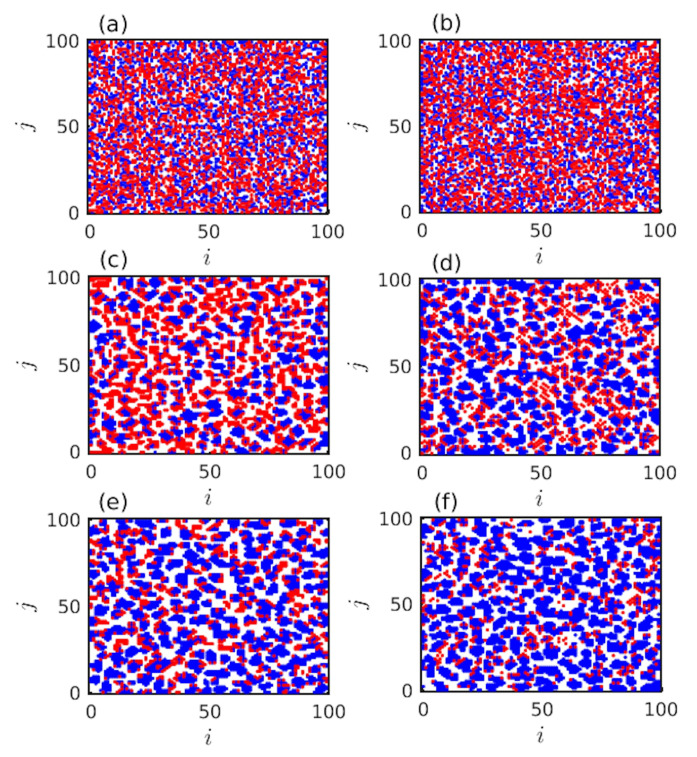
Effect of *r*: $T_{SD}=T_{PD}=1.3$ for both layers and other parameters are same as mentioned in the text. Initially 50% cooperators and 50% defectors are considered in the L×L lattices with $f_{0}=50\%$ and L=100. (**a**,**b**) Initial snapshots at t=0, (**c**,**d**) snapshots at $t=10^{3}$ with r=0, and (**e**,**f**) snapshots at $t=10^{3}$ with r=0.2. The left panel corresponds to the PD layer while the right panel shows simulations for the SD layer. Blue and red respectively represents the cooperators and defectors. White colors signify the free spaces. The second row represents two independent networks (as r=0), where the global average is 65.69%. Global average at r=0.2 (see the third row) is $f_{c}=83.32\%$, when the global network reaches its stationary fraction of cooperation. These snapshots represent the fact that a small enhancement of interdependence parameter *r* leads to an impressive improvement of the fraction of cooperation $f_{c}$ in both layers.

**Figure 4:**
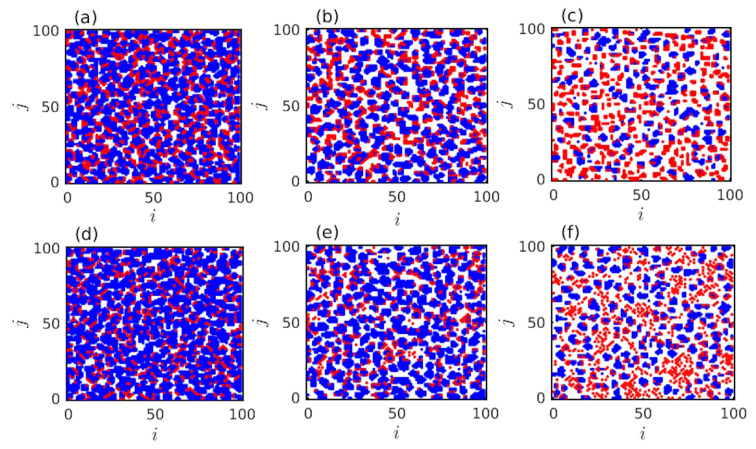
Effect of free space $f_{0}$: Parameters are same as mentioned in Figure 3. Here, *r* is kept fixed at 0.2. Snapshots taken at the final time iteration t=1000, for (**a**,**d**) $f_{0}=0.3$, (**b**,**e**) $f_{0}=0.5$, and (**c**,**f**) $f_{0}=0.7$, respectively. The upper panel and the lower panel shows the results for PD and SD layers, respectively. Increment of $f_{0}$ reduces the fraction of cooperation in both the layers.

Note that, although $f_{c}$ achieves a time-independent stationary state for both layers as per our numerical simulations (Figure 2), but the spatiotemporal structures are not static with respect to time. Not only the size, but also the shape of the clusters are changing with respect to time. To demonstrate this feature, few snapshots are plotted in Figure 3 and Figure 4. Figure 3 reveals that a slight increment in *r* with appropriate choices of other parameters helps to construct numerous cooperative (blue) clusters and hence, increases the $f_{c}$ for both layers to a surprising degree. In Figure 3a,b, the fraction of cooperation $f_{c}$ in both layers are 50%, as those figures are snapshots at initial time t=0. In Figure 3c,d, the snapshots are shown at time $t=10^{3}$ with r=0. Hence, those snapshots represent two independent networks. The simulations reveal as per those specific snapshots, that the final fraction of cooperation in the PD layer is $f_{c}=55.52\%$ and $f_{c}=75.86\%$ for the SD layer. All the simulations are performed in Figure 3 with L=100 and $f_{0}=50\%$. A notable change is observed in Figure 3e,f, where r=0.2 is taken. Thus, as the global network becomes interdependent, $f_{c}$ is significantly enhanced in both layers. Here we observe, $f_{c}=78.72\%$ for the PD layer and $f_{c}=87.92\%$ for the SD layer. This attests the influence of interdependence parameter *r* in our study.

The effect of free spaces are portrayed in Figure 4. Our simulations identify that introduction of sufficient amount of $f_{0}$, with suitable choices of other parameters, helps to suppress the competition for resources. The mobility of the players tends to overcome the inhibiting factors and thus, helps to increase cooperation on average. Here, L=100 and r=0.2 are kept fixed. We fix $f_{0}=30\%$ in Figure 4a,d. For this choice of proportion of free spaces, we find the fraction of cooperation $f_{c}=84.1714\%$ for PD layer (see Figure 4a) and $f_{c}=90.8429\%$ for the SD layer (see Figure 4d). In the third column of the Figure 4 (see Figure 4c,f), we set $f_{0}=0.7$ and we find $f_{c}=51.4333\%$ for PD layer and $f_{c}=68.2\%$ for the SD layer. Thus, although for $f_{0}=0.7$, the fraction of cooperation is increased for both layers compared to the initial $f_{c}$, as the initial $f_{c}$ is kept fixed at 50%. But, this increment is smaller compared to the rate of enhancement of $f_{c}$ for $f_{0}=0.3$. In the middle column of Figure 4, we set the free spaces at an intermediate value $f_{0}=50\%$. For this choice, $f_{c}$ is found to be 78.72% for the PD layer (see Figure 4b) and 87.92% for the SD layer (see Figure 4e), respectively. These snapshots at Figure 4 suggest the role of proportion of free spaces $f_{0}$ in our simulations.

Better opportunity based on the local interactions will always lead to change in their corresponding strategies. To improve $f_{c}$, our proposed migration and imitation strategy does not need any memory of past iterations. Even we do not introduce any kind of cost among the defectors [71]. Still initially randomly spread defectors are avoided by the cooperative neighbors. Those individuals can interact with any individuals. Even, the number of interactions among those individuals may interact only once, but there are still fair chances that they will interact more than once. Figure 3 and Figure 4 demonstrate the defectors ultimately form clusters or end up at the boundaries of the cooperative groups. Since the cooperators in the cluster have a tendency of having more successful neighbors, the defectors at the boundary of the cooperative cluster become cooperators. This reduces the average payoffs of defectors and consequently helps to promote cooperation. Though the defectors do not extinct as cooperative clusters are continuously interacted by defectors.

**Figure 5:**
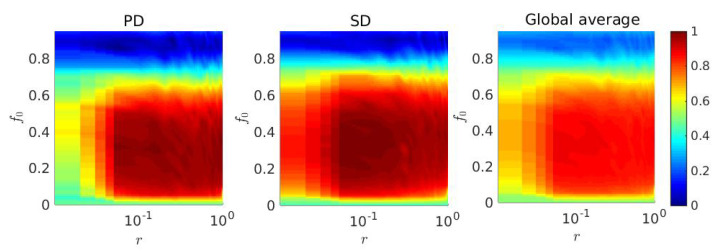
Fraction of cooperation in the parameter space $\left(r,f_{0}\right)$: Here, $T_{PD}=1.3=T_{SD}$. The fraction of free-space, $f_{0}$ is studied within [0%,95%]. Also, r∈(0.0,1.0]. Introduction of a tiny amount $r-f_{0}$ can outperform the defectors in both layers and as a result of that, the number of defectors are dimished to a significant level. To establish the improvement of fraction of cooperation on the global network, we plot the global average (right panel) along with the fraction of cooperation of PD layer (left panel) and of SD layer (middle panel).

To further inspect the effect of *r* and $f_{0}$ in our proposed model, a two-dimensional parameter space is plotted with respect to $f_{c}$. When r=1.0, each individual can only update its strategy from occupied (non-empty) replica node of the neighbor layer through the directed interlink. While r=0.0 implies *M* isolated independent layers of two different games. Whereas, $f_{0}=0$ demonstrates the scenario where each layer is filled with exactly N×N number of individuals with L=N=100 and hence, there is no possibility of migration in the lattice. Under these circumstances, individuals can not migrate into any neighboring cells and thus, they can not update their strategy. Hence, the final $f_{c}$ for $f_{0}=0$ will always reflect the initial setup in our proposed model. On the other hand, 100% free space, i.e., $f_{0}=1.0$ signifies the entire lattice is free from any individuals. Our simulations (Figure 5) suggest that there exists an optimal range for which $f_{c}$ will be maximized in both layers.

**Figure 6:**
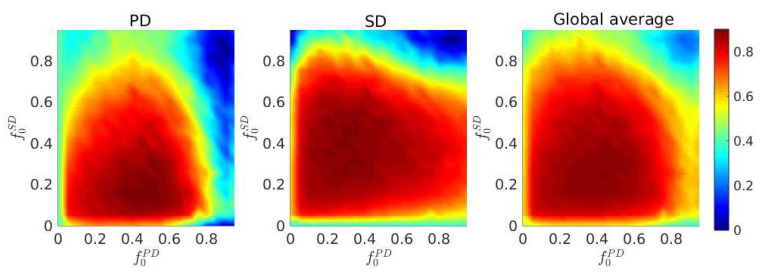
Fraction of cooperation $f_{c}$ as a function of $f_{0}^{PD}$ and $f_{0}^{SD}$: the effect of different proportion of free spaces on different layers is inspected here in order to maximize $f_{c}$ in both layers. The left plot is for the PD layer, whereas the middle panel is for the SD layer, and global average is plotted in the right panel. Here, r=0.2 and $T_{PD}=1.3=T_{SD}$. $f_{c}$ is increased notably for intermediate choices of $f_{0}^{PD}$ and $f_{0}^{SD}$.

Even, the interplay of different fractions of free spaces in the two layers are examined by keeping fixed the probability at r=0.2. Although, all other simulations in our study were done by keeping the same fraction of free spaces (i.e., $f_{0}^{PD}=f_{0}^{SD}=f_{0}$) in all the layers, but Figure 6 indicates there too exists a certain favourable choices of $\left(f_{0}^{PD},f_{0}^{SD}\right)$ which will enhance $f_{c}$ significantly in all layers. It is noticeable that the proportion of cooperators is maximized in an intermediate range of population densities. As $f_{0}$ of any layer tends to 0+, lack of migration opportunities hinders the maximization of $f_{c}$ in that layer. In the low density of population ($f_{0}\to 1-$), individuals will not be benefited by our algorithm as it is hard to find any neighbor, on average, at that circumstances.

**Figure 7:**
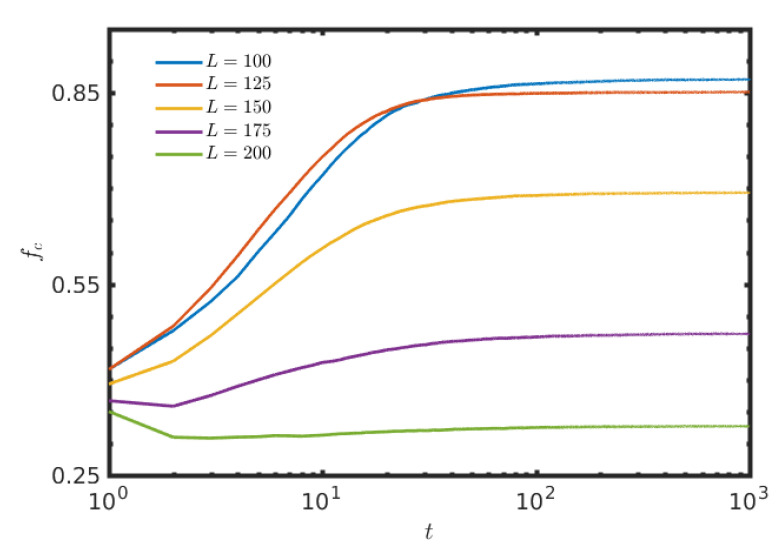
Effect of increment of lattice size keeping the total number of individuals unchanged: The size of the square lattice *L* is varied as shown in the figure. For all time series, initially at time t=0, the number of cooperators is 4000 and the number of defectors is kept fixed at 4000. The other parameters are r=0.2 and $T_{PD}=1.3=T_{SD}$. Since, the total number of individuals is initially fixed at t=0, so the increment of lattice size gives those individuals more free spaces to roam. The figure clearly illustrates the inclusion of more free spaces, by keeping the total number of individuals unchanged, actually decreases the fraction of cooperation $f_{c}$. Here, the global average (i.e., the average of the fraction of cooperation of both layers) is plotted with respect to *t*. Note that, the *x*-axis is in the logarithmic scale, so the initial data at t=0 is not incorporated.

Till now, the results are represented only by varying $f_{0}$ of both layers, but the size of the lattice remains unchanged. But, such a variation of $f_{0}$ with fixed *L* leads to a fluctuation in the effective population densities in both layers. To study the consequences of varying free spaces in another way, we plot the Figure 7 by changing the size *L* of the square lattices. The total number of individuals, N=8000, initially kept fixed at time t=0. The initial fraction of cooperation in all the cases in Figure 7 is $f_{c}=0.5$. Since, the initial effective population size is kept fixed, thus by changing *L*, one can study the effect of free-space, $f_{0}$. The size of the lattice is varied within [100,200] with a fixed step-length 25. As a result of that, the fraction of free positions, $f_{0}$ is also varied within [0.2,0.8]. The results depict the fact that, as the fraction of free position is increased with a fixed initial population, the fraction of cooperation is also decreased. Actually, as the lattice size increases, each individual gets more opportunity to move, but lessens their scope to interact with others due to the absence of a sufficient number of players in their neighborhood, on average. This hinders the growth of the enhancement of $f_{c}$ in both the layers. To portray the global scenario, the global average is plotted. Note that, the figure is plotted on a semi-log scale, hence the initial $f_{c}$ at t=0 is not shown there. The time series in the Figure 7 exhibits the similar notch type behavior at around t=3, as already observed in the Figure 2.

**Figure 8:**
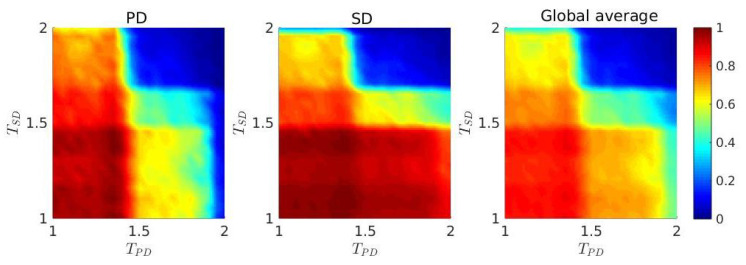
Effect of temptation parameter *T*: $f_{0}=50\%$ and r=0.2. Frequency of cooperators, $f_{c}$ is treated here as a function of the advantage of defectors, *T*. $T_{PD}$ denotes the temptation for the PD layer and temptation for the SD layer is represented as $T_{SD}$. Cooperation is enhanced for an optimal range of $\left[T_{PD},T_{SD}\right]$, where $T_{PD}$ and $T_{SD}$ both belongs to the range [1.0,1.4), approximately. The color bar indicates $f_{c}$. Imitating a neighbor from the SD layer (middle panel) is beneficial for the levels of cooperation in the PD layer (left panel) for $T_{PD}<1.4$ and $T_{SD}<1.4$. This fact is portrayed through the right panel of the figure, where we plot the global average. Note that, the fraction of cooperators is, in general, lower in the PD layer compared to the SD layer.

Temptation to defect always affects the dynamical behavior of the system. The interplay between $T_{PD}$ and $T_{SD}$ can lead the system from one desired dynamical regime to another undesired one. We numerically observe in Figure 8, that there exists a regime for 1≤T<1.4 (approximately) for both layers, which notably improves $f_{c}$ in all layers. An analytic understanding of the phenomenon of persistence and dominance of cooperation induced by our migration and stochastic imitation strategy appears difficult at this time. We thus seek to explain the phenomenon qualitatively, with the aid of numerical simulations. The two-dimensional parameter space of $T_{PD}$ and $T_{SD}$ for the entire interdependent network clearly delineates the fact that $T_{PD}<1.4$ and $T_{SD}<1.4$ promote the evolution of cooperation more effectively. This may be due to the accumulated payoffs in the SD layer. In a Von Neumann neighborhood surrounded by the equal number of cooperators and defectors, a cooperator will receive n(R+S) for n=1,2, which is equal to *n*, for our choice of parameter values in both layers. On the other hand, a defector will gain n(P+T) for n=1,2 on an evenly composed neighborhood of cooperators and defectors. For our chosen values $R_{PD}=R_{SD}=1.0$, $S_{PD}=S_{SD}=0.0$, $P_{PD}=0.1$ and $P_{SD}=-0.4$, the relation $n\left(R_{PD}+S_{PD}\right)<n\left(P_{PD}+T_{PD}\right)$ for n=1,2 holds always. But for the SD layer in a position surrounded by the same number of C’s and D’s, we have

$$\left\{\begin{array}{cccc} n\left(R_{SD}+S_{SD}\right) & > & n\left(P_{SD}+T_{SD}\right) & T_{SD}<1.4\\ & = & n(P_{SD}+T_{SD}) & T_{SD}=1.4\\ & < & n(P_{SD}+T_{SD}) & T_{SD}>1.4 \end{array}\right.$$

with n=1,2.

Under these circumstances, defectors of the SD layer cannot gain enough profits from neighbors for T<1.4 and thus become vulnerable to cooperators. As a result of that, cooperators tend to dominate eventually. Hence, up to T<1.4, SD layer can help in the survival of cooperative behavior based on our strategy and thus, eventually promotes the reduction of defectors on the global interdependent network.

**Figure 9:**
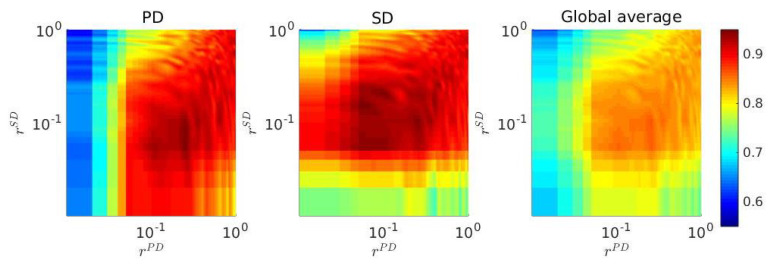
Consequences of various $r^{PD}$ and $r^{SD}$ on $f_{c}$: color-coded $f_{c}$ for the PD game (**left**), SD game (**middle**), and global average (**right**) on the $r^{PD}-r^{SD}$ parameter space is plotted based on the stationary fraction of cooperation. Here, $f_{0}=50\%$ and $T_{PD}=T_{SD}=1.3$. Note that both scales are logarithmic. Clearly, coooperation is best promoted, when interdependence parameters $r^{PD}$ and $r^{SD}$ are chosen from an optimal range.

Note that the parameter *r* here is designated as network interdependence parameter. Our proposed strategy updating protocol only allows the players to interact in their local neighborhood, but occasionally they can update their strategy from one of the long distant neighbors on the other network with probability *r*. Every player can only connect with their replica player, provided the replica position in the other network is not empty. To understand the role of this network interdependence, a two-dimensional parameter space (Figure 9) is drawn in the $\left(r^{PD},r^{SD}\right)$ space, by considering different strategy updating probabilistic fractions in both layers. Clearly, there exists an optimal region of intermediate interdependence between the two layers, which enhances the cooperation on both networks. Our theoretical simulation suggests a suitable choices of parameter *r* can drastically maximize the fraction of cooperation for both layers. Besides our movement strategy, this random strategy adaptation is found to intensify the cooperation level, on average.

## 4. Discussion

Coevolutionary processes on interdependent networks provide a rich playground that can be implemented successfully on various topics that are of relevance to social sciences, as well as to natural sciences and engineering, ranging from traffic [72], crime [73], epidemic processes [74], climate inaction [75], antibiotic overuse [76], and vaccination [77,78], which can be put under the umbrella of social physics [79].

Along these lines, our research reveals the role of goal-oriented migration in an interdependent network, where individuals on two different layers are playing two distinct games, namely the prisoner’s dilemma and the snowdrift game. Earlier studies on multilayer networks [67,68] uncovered that interdependence between networks generally inhibits the cooperation in the SD layer, although it is found to be useful for PD layer. In contrast, our mobility induced strategy improves the level of cooperation significantly in both layers. Each individual has been treated here as a mobile agent, looking for a better neighborhood in order to maximize the profit in terms of payoffs. This mobility influences the population dynamics and facilitates cooperators to survive appreciably by evading the invasions by defectors. Our systematic simulations indicate that the success of cooperators is enhanced in an intermediately dense population, where the availability of free space is sufficient. Furthermore, we have shown that the performance of the proposed strategy will increase under suitable choices of the interdependence parameter and for a suitable value of the temptation parameter within the [1.0,1.4) range. We have also studied the effect of free spaces by keeping the effective population size unchanged. We have also applied our approach to a multilayer network where on both layers the same game model was applied, and we have likewise observed a notable enhancement of cooperation (results not shown here).

The considered migration-induced interaction dynamics may lead to an interesting direction for future research at the interface of multilayer networks and evolutionary game theory, in particular with the aim of engineering better social systems at the interface of networks and human decision making under social dilemma conditions.

## References

[1] Sigmund K. (2010). The Calculus of Selfishness.

[2] Perc M., Grigolini P. (2013). Collective behavior and evolutionary games-an introduction. Chaos Solitons Fract.

[3] Nowak M.A. (2006). Five rules for the evolution of cooperation. Science.

[4] Szolnoki A., Mobilia M., Jiang L.L., Szczesny B., Rucklidge A.M., Perc M. (2014). Cyclic dominance in evolutionary games: A review. J. R. Soc. Interface.

[5] Weibull J.W. (1995). Evolutionary Game Theory.

[6] Perc M., Szolnoki A. (2010). Coevolutionary games–A mini review. BioSystems.

[7] Capraro V., Perc M. (2018). Grand challenges in social physics: In pursuit of moral behavior. Front. Phys..

[8] Chowdhury S.N., Majhi S., Ozer M., Ghosh D., Perc M. (2019). Synchronization to extreme events in moving agents. New. J. Phys..

[9] Axelrod R. (1984). The Evolution of Cooperation.

[10] Skutch A.F. (1961). Helpers among birds. Condor.

[11] Wang R.W., Shi L., Ai S.M., Zheng Q. (2008). Trade-off between reciprocal mutualists: Local resource availability-oriented interaction in fig/fig wasp mutualism. J. Anim. Ecol..

[12] Wilson E.O. (1971). The Insect Societies.

[13] Axelrod R., Hamilton W.D. (1981). The evolution of cooperation. Science.

[14] Smith J.M., Price G.R. (1973). The logic of animal conflict. Nature.

[15] Santos F.C., Pacheco J.M. (2005). Scale-free networks provide a unifying framework for the emergence of cooperation. Phys. Rev. Lett..

[16] Santos F.C., Pacheco J.M., Lenaerts T. (2006). Evolutionary dynamics of social dilemmas in structured heterogeneous populations. Proc. Natl. Acad. Sci. USA.

[17] Poncela J., Gómez-Gardeñes J., Floría L.M., Moreno Y. (2007). Robustness of cooperation in the evolutionary prisoner’s dilemma on complex systems. New J. Phys..

[18] Gómez-Gardenes J., Campillo M., Floría L.M., Moreno Y. (2007). Dynamical organization of cooperation in complex topologies. Phys. Rev. Lett..

[19] Antonioni A., Tomassini M. (2011). Network Fluctuations Hinder Cooperation in Evolutionary Games. PLoS ONE.

[20] Tanimoto J., Brede M., Yamauchi A. (2012). Network reciprocity by coexisting learning and teaching strategies. Phys. Rev. E.

[21] Antonioni A., Cacault M.P., Lalive R., Tomassini M. (2014). Know Thy Neighbor: Costly Information Can Hurt Cooperation in Dynamic Networks. PLoS ONE.

[22] Wang Z., Kokubo S., Jusup M., Tanimoto J. (2015). Universal scaling for the dilemma strength in evolutionary games. Phys. Life Rev..

[23] Javarone M.A. (2016). Statistical physics of the spatial Prisoner’s Dilemma with memory-aware agents. Eur. Phys. J. B.

[24] Amaral M.A., Javarone M.A. (2018). Heterogeneous update mechanisms in evolutionary games: Mixing innovative and imitative dynamics. Phys. Rev. E.

[25] Vilone D., Capraro V., Ramasco J.J. (2018). Hierarchical invasion of cooperation in complex networks. J. Phys. Commun..

[26] Fotouhi B., Momeni N., Allen B., Nowak M.A. (2019). Evolution of cooperation on large networks with community structure. J. R. Soc. Interface.

[27] Boccaletti S., Latora V., Moreno Y., Chavez M., Hwang D.U. (2006). Complex networks: Structure and dynamics. Phys. Rep..

[28] Barabási A.L. (2015). Network Science.

[29] Estrada E. (2012). The Structure of Complex Networks: Theory and Applications.

[30] Barrat A., Barthélemy M., Vespignani A. (2008). Dynamical Processes on Complex Networks.

[31] Nowak M.A., May R.M. (1992). Evolutionary games and spatial chaos. Nature.

[32] Szabó G., Fath G. (2007). Evolutionary games on graphs. Phys. Rep..

[33] Kivelä M., Arenas A., Barthelemy M., Gleeson J.P., Moreno Y., Porter M.A. (2014). Multilayer networks. J. Complex Netw..

[34] Boccaletti S., Bianconi G., Criado R., Del Genio C.I., Gómez-Gardenes J., Romance M., Sendina-Nadal I., Wang Z., Zanin M. (2014). The structure and dynamics of multilayer networks. Phys. Rep..

[35] Majhi S., Perc M., Ghosh D. (2017). Chimera states in a multilayer network of coupled and uncoupled neurons. Chaos.

[36] Kundu S., Majhi S., Ghosh D. (2019). From asynchronous to synchronous chimeras in ecological multiplex network. Eur. Phys. J. Spec. Top..

[37] Majhi S., Perc M., Ghosh D. (2016). Chimera states in uncoupled neurons induced by a multilayer structure. Sci. Rep..

[38] Rakshit S., Majhi S., Bera B.K., Sinha S., Ghosh D. (2017). Time-varying multiplex network: Intralayer and interlayer synchronization. Phys. Rev. E.

[39] Kundu S., Majhi S., Ghosh D. (2019). Chemical synaptic multiplexing enhances rhythmicity in neuronal networks. Nonlinear Dyn..

[40] Wang Z., Wang L., Szolnoki A., Perc M. (2015). Evolutionary games on multilayer networks: A colloquium. Eur. Phys. J. B.

[41] Buldyrev S.V., Parshani R., Paul G., Stanley H.E., Havlin S. (2010). Catastrophic cascade of failures in interdependent networks. Nature.

[42] Wang Z., Wang L., Perc M. (2014). Degree mixing in multilayer networks impedes the evolution of cooperation. Phys. Rev. E.

[43] Szolnoki A., Perc M. (2009). Emergence of multilevel selection in the prisoner’s dilemma game on coevolving random networks. New. J. Phys..

[44] Duh M., Gosak M., Slavinec M., Perc M. (2019). Assortativity provides a narrow margin for enhanced cooperation on multilayer networks. New. J. Phys..

[45] Szolnoki A., Perc M. (2009). Promoting cooperation in social dilemmas via simple coevolutionary rules. Eur. Phys. J. B.

[46] Szolnoki A., Szabó G. (2007). Cooperation enhanced by inhomogeneous activity of teaching for evolutionary Prisoner’s Dilemma games. EPL Europhys. Lett..

[47] Perc M., Wang Z. (2010). Heterogeneous aspirations promote cooperation in the prisoner’s dilemma game. PLoS ONE.

[48] Szolnoki A., Perc M., Szabó G. (2008). Diversity of reproduction rate supports cooperation in the prisoner’s dilemma game on complex networks. Eur. Phys. J. B.

[49] Wang Z., Perc M. (2010). Aspiring to the fittest and promotion of cooperation in the prisoner’s dilemma game. Phys. Rev. E.

[50] Szabó G., Szolnoki A. (2009). Cooperation in spatial prisoner’s dilemma with two types of players for increasing number of neighbors. Phys. Rev. E.

[51] Zhu C.J., Sun S.W., Wang L., Ding S., Wang J., Xia C.Y. (2014). Promotion of cooperation due to diversity of players in the spatial public goods game with increasing neighborhood size. Phys. A Stat. Mech. Appl..

[52] Helbing D., Yu W. (2009). The outbreak of cooperation among success-driven individuals under noisy conditions. Proc. Natl. Acad. Sci. USA.

[53] Jiang L.L., Wang W.X., Lai Y.C., Wang B.H. (2010). Role of adaptive migration in promoting cooperation in spatial games. Phys. Rev. E.

[54] Meloni S., Buscarino A., Fortuna L., Frasca M., Gómez-Gardeñes J., Latora V., Moreno Y. (2009). Effects of mobility in a population of prisoner’s dilemma players. Phys. Rev. E.

[55] Noh J.D., Rieger H. (2004). Random walks on complex networks. Phys. Rev. Lett..

[56] Aktipis C.A. (2004). Know when to walk away: Contingent movement and the evolution of cooperation. J. Theor. Biol..

[57] Vainstein M.H., Silva A.T., Arenzon J.J. (2007). Does mobility decrease cooperation?. J. Theor. Biol..

[58] Smaldino P.E., Schank J.C. (2012). Movement patterns, social dynamics, and the evolution of cooperation. Theor. Popul. Biol..

[59] Tanimoto J., Sagara H. (2007). Relationship between dilemma occurrence and the existence of a weakly dominant strategy in a two-player symmetric game. BioSystems.

[60] Smith J.M. (1982). Evolution and the Theory of Games.

[61] Sugden R. (1986). The Economics of Rights, Cooperation and Welfare.

[62] Tayer M. (1987). Possibility of Cooperation: Studies in Rationality and Social Change.

[63] Scheuring I. (2005). The iterated continuous prisoner’s dilemma game cannot explain the evolution of interspecific mutualism in unstructured populations. J. Theor. Biol..

[64] Tanimoto J. (2009). A simple scaling of the effectiveness of supporting mutual cooperation in donor-recipient games by various reciprocity mechanisms. BioSystems.

[65] Berger U. (2009). Simple scaling of cooperation in donor-recipient games. BioSystems.

[66] Hauert C., Doebeli M. (2004). Spatial structure often inhibits the evolution of cooperation in the snowdrift game. Nature.

[67] Santos M.D., Dorogovtsev S.N., Mendes J.F. (2014). Biased imitation in coupled evolutionary games in interdependent networks. Sci. Rep..

[68] Wang B., Pei Z., Wang L. (2014). Evolutionary dynamics of cooperation on interdependent networks with the Prisoner’s Dilemma and Snowdrift Game. EPL Europhys. Lett..

[69] Wang Z., Szolnoki A., Perc M. (2013). Optimal interdependence between networks for the evolution of cooperation. Sci. Rep..

[70] Gómez-Gardenes J., Reinares I., Arenas A., Floría L.M. (2012). Evolution of cooperation in multiplex networks. Sci. Rep..

[71] Banerjee J., Layek R.K., Sasmal S.K., Ghosh D. (2019). Delayed evolutionary model for public goods competition with policing in phenotypically variant bacterial biofilms. EPL Europhys. Lett..

[72] Helbing D. (2001). Traffic and related self-driven many-particle systems. Rev. Mod. Phys..

[73] D’Orsogna M.R., Perc M. (2015). Statistical physics of crime: A review. Phys. Life Rev..

[74] Pastor-Satorras R., Castellano C., Van Mieghem P., Vespignani A. (2015). Epidemic processes in complex networks. Rev. Mod. Phys..

[75] Pacheco J.M., Vasconcelos V.V., Santos F.C. (2014). Climate change governance, cooperation and self-organization. Phys. Life Rev..

[76] Chen X., Fu F. (2018). Social learning of prescribing behavior can promote population optimum of antibiotic use. Front. Phys..

[77] Fu F., Rosenbloom D.I., Wang L., Nowak M.A. (2011). Imitation dynamics of vaccination behaviour on social networks. Proc. R. Soc. B.

[78] Wang Z., Bauch C.T., Bhattacharyya S., d’Onofrio A., Manfredi P., Perc M., Perra N., Salathé M., Zhao D. (2016). Statistical physics of vaccination. Phys. Rep..

[79] Perc M. (2019). The social physics collective. Sci. Rep..

